# ZBP1 promotes fungi-induced inflammasome activation and pyroptosis, apoptosis, and necroptosis (PANoptosis)

**DOI:** 10.1074/jbc.RA120.015924

**Published:** 2021-01-13

**Authors:** Balaji Banoth, Shraddha Tuladhar, Rajendra Karki, Bhesh Raj Sharma, Benoit Briard, Sannula Kesavardhana, Amanda Burton, Thirumala-Devi Kanneganti

**Affiliations:** Department of Immunology, St. Jude Children's Research Hospital, Memphis, Tennessee, USA

**Keywords:** cell death, inflammasome, PANoptosis, PANoptosome, pyroptosis, apoptosis, necroptosis, ZBP1, gasdermin D, MLKL, RIPK3, caspase-1, caspase-8, caspase-3, caspase-7, Aspergillus fumigatus, Candida albicans, fungi, infection, infectious disease, host defense

## Abstract

*Candida albicans and Aspergillus fumigatus* are dangerous fungal pathogens with high morbidity and mortality, particularly in immunocompromised patients. Innate immune-mediated programmed cell death (pyroptosis, apoptosis, necroptosis) is an integral part of host defense against pathogens. Inflammasomes, which are canonically formed upstream of pyroptosis, have been characterized as key mediators of fungal sensing and drivers of proinflammatory responses. However, the specific cell death pathways and key upstream sensors activated in the context of *Candida* and *Aspergillus* infections are unknown. Here, we report that *C. albicans* and *A. fumigatus* infection induced inflammatory programmed cell death in the form of pyroptosis, apoptosis, and necroptosis (PANoptosis). Further, we identified the innate immune sensor Z-DNA binding protein 1 (ZBP1) as the apical sensor of fungal infection responsible for activating the inflammasome/pyroptosis, apoptosis, and necroptosis. The Zα2 domain of ZBP1 was required to promote this inflammasome activation and PANoptosis. Overall, our results demonstrate that *C. albicans* and *A. fumigatus* induce PANoptosis and that ZBP1 plays a vital role in inflammasome activation and PANoptosis in response to fungal pathogens.

Mycotic diseases pose a significant global health burden, particularly among immunocompromised patients, and they are under studied compared with other infectious diseases (1). *Candida albicans* and *Aspergillus fumigatus* infections often result in invasive candidiasis and aspergilliosis if not cleared early, and they are among the most dangerous fungal pathogens with high morbidity and mortality rates (2, 3). The host innate immune system is critical for recognizing fungal particles and mediating their clearance (4). Innate immune-mediated programmed cell death (pyroptosis, apoptosis, necroptosis) is also an essential part of this host defense (5). Pathogen-associated molecular patterns and damage-associated molecular patterns (PAMPs and DAMPs) are recognized through pattern recognition receptors (PRRs) and can cause the formation of cytosolic multimeric protein complexes known as inflammasomes (6). The inflammasome drives the activation of the inflammatory caspase-1 (CASP1), which further proteolytically processes cytokines including interleukin (IL)-1β and IL-18 and the executioner molecule gasdermin D (GSDMD), resulting in pyroptotic cell death (6, 7, 8). Previously, we have characterized inflammasome sensors, including NLRP3 and AIM2, to mediate innate immune responses against fungal pathogens, *C. albicans* and *A. fumigatus* (9, 10, 11). Fungi-mediated inflammasome activation causes cytokine release and tissue damage because of inflammatory cell death (12). In addition to pyroptosis, other forms of programmed cell death important for host defense include apoptosis and necroptosis. Apoptosis is induced on activation of apical caspases caspase-8 (CASP8) or caspase-9 (CASP9) that results in activation of executioner caspases, including caspase-3 (CASP3) and caspase-7 (CASP7) (13, 14). The apoptotic caspase-3 can also activate gasdermin E to induce a lytic form of cell death (15). During necroptosis, proteins containing a receptor interacting protein (RIP) homotypic interaction motif (RHIM), including RIP kinase 1 (RIPK1) and RIP kinase 3 (RIPK3), play crucial roles in the phosphorylation of the mixed lineage kinase domain-like pseudokinase (MLKL), which executes cell death (13, 16).

The identification of extensive cross-talk between pyroptosis, apoptosis, and necroptosis has led to the establishment of the concept of “PANoptosis” (17, 18, 19, 20, 21, 22, 23, 24, 25, 26, 27, 28, 29, 30). PANoptosis (‘P’, Pyroptosis; ‘A’, Apoptosis; ‘N’, Necroptosis; and ‘optosis’, a form of programmed cell death) is defined as “a unique inflammatory programmed cell death regulated by the PANoptosome, which provides a molecular scaffold that allows for interactions and activation of the machinery required for inflammasome/pyroptosis (such as NLRP3, ASC, caspase-1), apoptosis (caspase-8), and necroptosis (RIPK3/RIPK1)” (17, 25, 28, 29). The ability of these molecules to interact allows for intricate coregulation between cell death pathways that had previously been thought to be independent. PANoptosis has been implicated in infectious and autoinflammatory diseases, cancer, and beyond (17, 18, 19, 20, 21, 23, 25, 26, 29, 30), and the molecular details and phenotypic outcomes of the cross-talk and coregulation among pyroptosis, apoptosis, and necroptosis are dependent on the stimulus provided.

In this study, we show that *C. albicans* and *A. fumigatus* infection elicited inflammasome activation and pyroptosis, apoptosis, and necroptosis (PANoptosis). We found that the innate immune sensor Z-DNA binding protein 1 (ZBP1) functioned as the apical sensor to activate the *C. albicans* and *A. fumigatus-*induced inflammasome/pyroptosis, apoptosis, and necroptosis. Further, we demonstrate that the nucleic acid binding domain Zα2 of ZBP1 is crucial for *C. albicans*- and *A. fumigatus*-induced inflammasome activation and PANoptotic cell death. Collectively, our results establish that *C. albicans* and *A. fumigatus* induce PANoptosis and that ZBP1 is critical for inflammasome activation and PANoptosis in response to fungal pathogens.

## Results

### C. albicans and A. fumigatus infections activate PANoptosis

**Figure 1:**
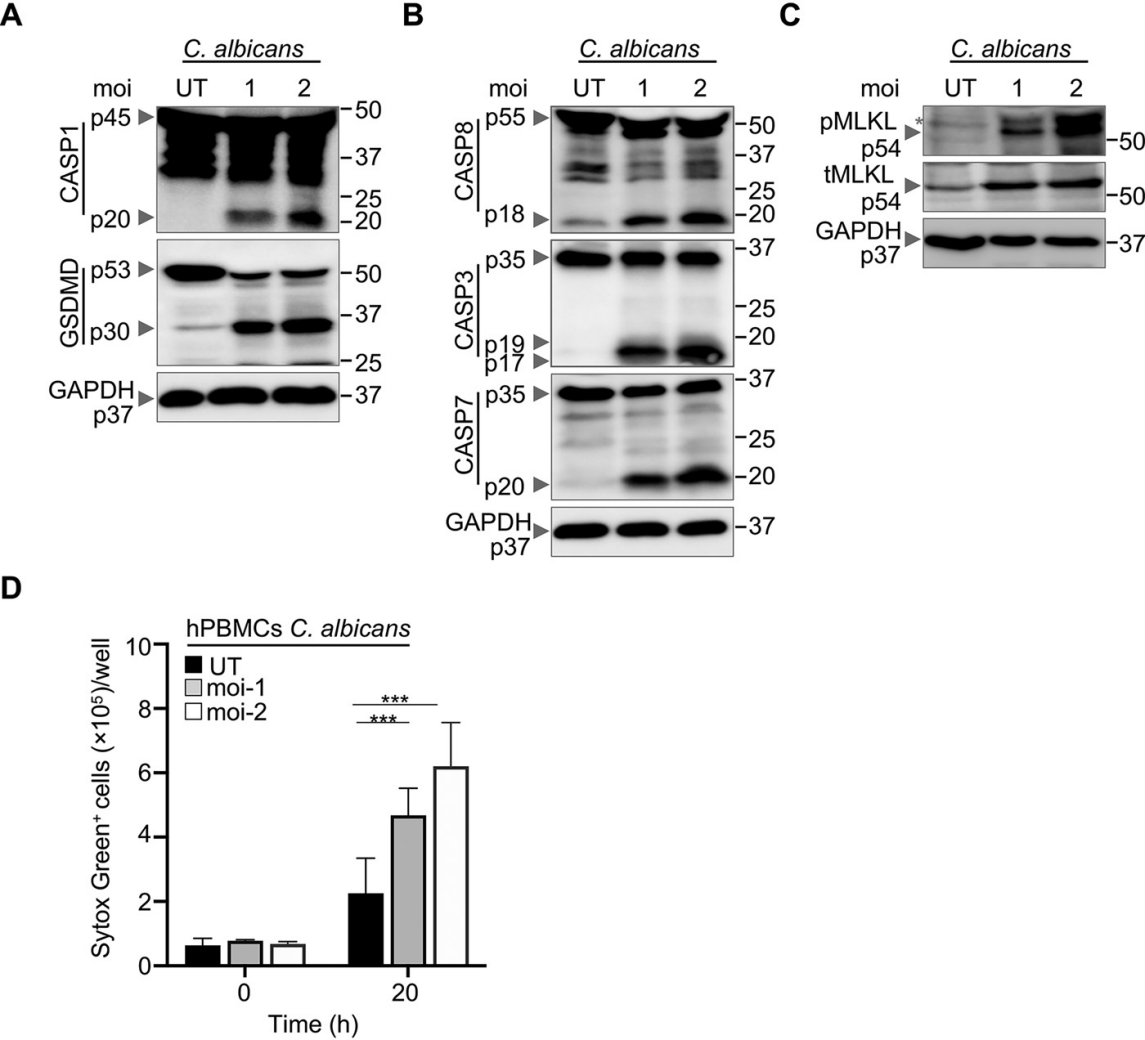
**PANoptosis in response to *C. albicans.****A*–*C*, Western blotting analysis of PANoptosis activation markers in bone marrow-derived macrophages (BMDMs) after *C. albicans* infection. *A*, Pyroptosis activation is assessed by immunoblotting of cleaved caspase-1 (CASP1) (p20) and gasdermin D (GSDMD) (p30). *B*, Apoptosis activation is determined by immunoblotting of active initiator CASP8 (p18) and executioner caspases CASP3 (p19/17) and CASP7 (p20). *C*, Necroptosis activation is indicated by the phosphorylation of mixed lineage kinase domain-like pseudokinase (pMLKL). Total MLKL (tMLKL) and GAPDH are used as loading controls. Molecular weight marker sizes are indicated on the right (kDa). *D*, Quantification of cell death in primary human peripheral blood mononuclear cells (hPBMCs) following *C. albicans* infection. Data presented are representative of three independent experiments. 2-way ANOVA was used to determine statistical significance. ***P < 0.001 *moi*, multiplicity of infection; *UT*, untreated. Black asterisks denote a nonspecific band.

Our previous studies characterized inflammasomes as key players in sensing fungal infection and activating proinflammatory responses (9, 10), but the specific cell death pathways activated in response to *C. albicans* and *A. fumigatus* remain unknown. Therefore, we systematically analyzed the programmed cell death pathways activated by *C. albicans* and *A. fumigatus*. We infected WT BMDMs with varying doses of *C. albicans* and *A. fumigatus* and biochemically assessed markers associated with pyroptosis, apoptosis, and necroptosis (PANoptosis). Consistent with previous reports (10), we found *C. albicans* and *A. fumigatus* elicited inflammasome activation and pyroptosis in a dose-dependent manner, as indicated by activation of CASP1 and GSDMD (Fig. 1*A*, Fig. S1). Further, characterization of cell death markers associated with apoptosis revealed increased activation of the apoptotic initiator CASP8 (p18) and executioners CASP3 (p17/19) and CASP7 (p20) in a dose-dependent manner (Fig. 1*B*, Fig. S1). The phosphorylated form of MLKL (pMLKL) is a marker of necroptosis activation (31). We observed that infection of WT BMDMs resulted in dose-dependent necroptotic cell death as evidenced by the enhanced levels of pMLKL (Fig. 1*C*, Fig. S1). Further, we also observed a dose-dependent induction of cell death in response to *C. albicans* infection in primary human peripheral blood mononuclear cells (hPBMCs), corroborating our findings in primary BMDMs (Fig. 1*D*). Overall, these data indicate that fungal infection with *C. albicans* and *A. fumigatus* activates PANoptosis.

### Loss of PANoptotic components confers protection against fungi-induced cell death

**Figure 2:**
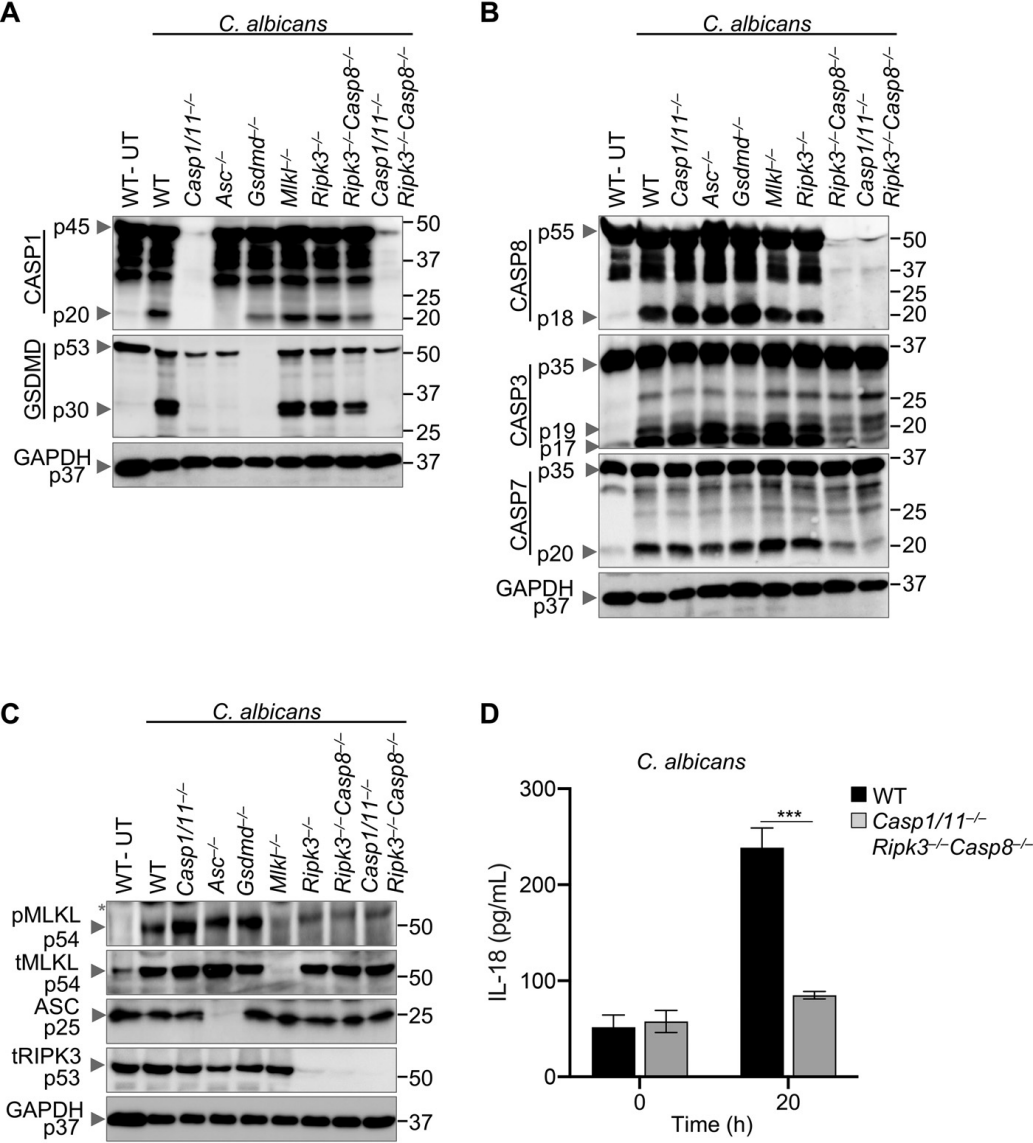
**Ablation of PANoptotic components inhibits *C. albicans*-induced PANoptosis and inflammation.***A*–*C*, Western blotting analysis of PANoptosis activation markers after *C. albicans* infection in the indicated bone marrow-derived macrophages (BMDMs). *A*, Pyroptosis activation is assessed by immunoblotting of cleaved caspase-1 (CASP1) (p20) and gasdermin D (GSDMD) (p30). *B*, Apoptosis activation is determined by immunoblotting of active initiator CASP8 (p18) and executioner caspases CASP3 (p19/17) and CASP7 (p20). *C*, Necroptosis activation is indicated by the phosphorylation of mixed lineage kinase domain-like pseudokinase (pMLKL). Total MLKL (tMLKL) and GAPDH are used as loading controls. ASC and RIPK3 proteins were probed to confirm their deletion in ASC and RIPK3-deficient BMDMs, respectively. Molecular weight marker sizes are indicated on the right (kDa). *D*, Inflammatory cytokine IL-18 release was evaluated in WT (WT) and *Casp1/11*^–/–^*Ripk3*^–/–^*Casp8*^–/–^ BMDMs following *C. albicans* infection for 20 h. Data shown are representative of at least three independent experiments (*A*–*D*). Unpaired *t* test with Welch's correction was used to determine statistical significance. ***P < 0.001 (*D*). *UT*, untreated. Black asterisks denote a nonspecific band.

Because we observed PANoptosis during fungal infection, we further evaluated the role of key molecular components of PANoptosis using a genetic approach. We infected murine BMDMs lacking crucial components of PANoptosis with *C. albicans* or *A. fumigatus* (Fig. 2, Fig. S2). Infection of WT BMDMs with *C. albicans* or *A. fumigatus* resulted in robust activation of proteins involved in pyroptosis, apoptosis, and necroptosis (Fig. 2*A*–*C*, Fig. S2A), as observed earlier (Fig. 1). Pyroptotic cell death is largely mediated by CASP1, CASP11, ASC, and GSDMD. Loss of these individual molecules led to reduced activation of pyroptotic proteins (CASP1 and GSDMD; Fig. 2*A*, Fig. S2A) and had minimal effect on the activation of apoptotic proteins (CASP8, CASP3, and CASP7; Fig. 2*B*, Fig. S2A); however, the expression of pMLKL was clearly increased during *C. albicans* infection and trended toward an increase during *A. fumigatus* infection (Fig. 2*C*, Fig. S2A), suggesting increased activation of necroptosis when pyroptosis is blocked in response to fungal pathogens. Deficiency of necroptotic mediators MLKL or RIPK3 had minor impacts on the activation of pyroptotic and apoptotic markers (Fig. 2*A*, *B*, Fig. S2A). CASP8 regulates both canonical and noncanonical NLRP3 inflammasome activation in bacterial and viral infections (17, 32), and it also negatively regulates the necroptotic pathway (14). BMDMs lacking both CASP8 and RIPK3 were largely protected from *C. albicans*- or *A. fumigatus*-induced PANoptosis, as evident by the reduced activation of pyroptotic (CASP1, GSDMD; Fig. 2*A*, Fig. S2A), apoptotic (CASP3, CASP7; Fig. 2*B*, Fig. S2A), and necroptotic (pMLKL) biochemical markers (Fig. 2*C*, Fig. S2A).

BMDMs lacking critical components of PANoptosis (CASP1, CASP11, RIPK3, and CASP8 combined) are protected from bacteria and virus–induced PANoptosis (17). Similar to the *Ripk3*^–/–^*Casp8*^–/–^ cells, *Casp1/11*^–/–^*Ripk3*^–/–^*Casp8*^–/–^ BMDMs showed reduced activation of host cell death markers in response to *C. albicans* or *A. fumigatus* infection (Fig. 2*A*–*C*, Fig. S2A). However, although *Ripk3*^–/–^*Casp8*^–/–^ cells showed residual levels of GSDMD activation, the activation of GSDMD was completely abolished in *Casp1/11*^–/–^*Ripk3*^–/–^*Casp8*^–/–^ BMDMs (Fig. 2*A*, Fig. S2A). Combined with the reduced activation of caspase-3, -7 and MLKL, these findings suggest cells undergo reduced cell death when crucial components of PANoptosis are missing during fungal infection.

Activation of the inflammasome and inflammatory cell death often results in the release of various cytokines, chemokines, and DAMPs which further amplify the inflammatory response and associated pathology (7, 33). We assessed inflammatory cytokine IL-18 release after *C. albicans* or *A. fumigatus* infection as a measure of inflammasome activation and associated cell death. WT BMDMs released a significant amount of IL-18 after fungal infection, whereas the *Casp1/11*^–/–^*Ripk3*^–/–^*Casp8*^–/–^ BMDMs released significantly less (Fig. 2*D*, Fig. S2B). Together, these findings suggest that the components of PANoptosis are crucial in mediating inflammatory cell death and cytokine release during fungal infection.

### ZBP1 regulates PANoptosis and inflammation via its Zα2 domain

**Figure 3:**
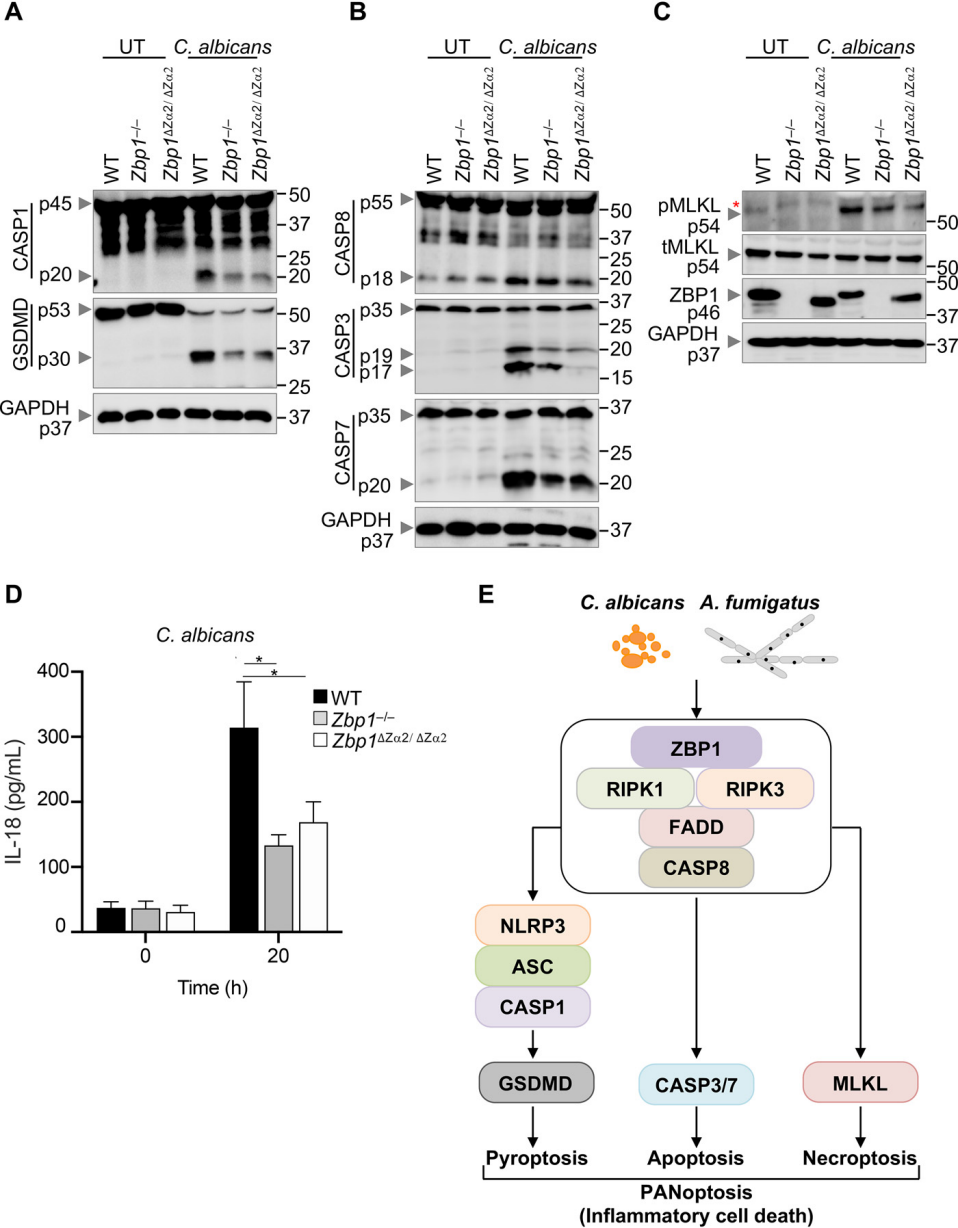
**Zα2 domain of ZBP1 drives PANoptosis.***A*–*C*, Western blot analysis of PANoptosis activation markers after *C. albicans* infection in wildtype (WT), *Zbp1*^–/–^, or *Zbp1*^ΔZα2/ΔZα2^ bone marrow-derived macrophages (BMDMs). *A*, Pyroptosis activation is assessed by immunoblotting of cleaved caspase-1 (CASP1) (p20) and gasdermin D (GSDMD) (p30). *B*, Apoptosis activation is determined by immunoblotting of active initiator CASP8 (p18) and executioner caspases CASP3 (p19/17) and CASP7 (p20). *C*, Necroptosis activation is indicated by the phosphorylation of mixed lineage kinase domain-like pseudokinase (pMLKL). ZBP1 protein was probed to confirm its deletion and MW shift in ZBP1-deficient and *Zbp1*^ΔZα2/ΔZα2^ BMDMs, respectively. Total MLKL (tMLKL) and GAPDH are used as loading controls. Molecular weight marker sizes are indicated on the right (kDa). *D*, Inflammatory cytokine IL-18 release was evaluated in WT, *Zbp1*^–/–^, or *Zbp1*^ΔZα2/ΔZα2^ BMDMs following *C. albicans* infection for 20 h. *E*, Schematic summary of the regulation of PANoptosis by ZBP1 in response to the fungal pathogens *C. albicans* or *A. fumigatus*. ZBP1 senses the fungal pathogen and mediates PANoptosis possibly by engaging the ZBP1-PANoptosome to drive NLRP3-mediated CASP1 activation (pyroptosis), CASP3/CASP7 activation (apoptosis), and MLKL activation (necroptosis). Data shown are representative of at least three independent experiments (*A*–*D*). 2-way ANOVA was employed to determine statistical significance. *P < 0.05 (*D*). UT, untreated. Red asterisks denote a nonspecific band.

The interferon (IFN)-inducible protein ZBP1, also known as DAI (DNA-dependent activator of IFN regulatory factors), is an innate immune sensor that mediates NLRP3 inflammasome activation in response to influenza A virus (IAV) infection (21, 29). ZBP1 contains two N-terminal nucleic acid binding domains (Zα1 and Zα2) followed by a RHIM domain responsible for mediating homotypic interactions with cell death signaling proteins RIPK1 and RIPK3 (20, 41). In the context of IAV, ZBP1 sensing of the virus leads to PANoptosome assembly and drives pyroptosis through NLRP3 inflammasome activation, apoptosis via FADD-CASP8, and necroptosis through RIPK3-MLKL (17, 21, 29, 34, 35, 36, 37), with CASP6 promoting the association between ZBP1 and RIPK3 (29). However, the role of ZBP1 in inflammasome activation and PANoptosis in response to other pathogens and fungi is not known. To understand the contribution of ZBP1 to PANoptosis during fungal infections, we infected BMDMs from WT and ZBP1-deficient mice with *C. albicans* or *A. fumigatus*. The WT BMDMs showed robust activation of the inflammasome and PANoptosis on infection with *C. albicans* or *A. fumigatus* (Fig. 3*A*–*C*, Fig. S3A). BMDMs deficient in ZBP1 (*Zbp1*^–/–^ cells) had reduced activation of the inflammasome and pyroptosis, as shown by the reduced cleavage of CASP1 and GSDMD (Fig. 3*A*, Fig. S3A). The activation of apoptosis and necroptosis as measured by the cleavage of CASP8, CASP3, and CASP7 and the level of pMLKL were also abrogated in the ZBP1-deficient BMDMs (Fig. 3*B*, *C*, Fig. S3A), and the changes in pMLKL were more notable with *C. albicans* than *A. fumigatus*. Thus, our results show a crucial role for the master regulator ZBP1 in controlling fungi-induced inflammasome activation and PANoptosis.

To further understand how ZBP1 mediated the activation of PANoptosis, we examined the role of the Zα2 domain in this process. Recently, the Zα2 domain was shown to act as a molecular switch in regulating IAV-induced NLRP3 inflammasome activation and PANoptosis (20). The Zα2 domain of ZBP1 senses endogenous and IAV-derived nucleic acids to mediate cell death and inflammation (20, 38), but whether it can do the same with fungal ligands is unknown. We infected WT and *Zbp1*^ΔZα2/ΔZα2^ BMDMs with *C. albicans* or *A. fumigatus* and biochemically assessed markers of PANoptosis. Inflammasome activation occurred in WT BMDMs, indicated by CASP1 and GSDMD cleavage, but their levels were substantially reduced in *Zbp1*^–/–^ and *Zbp1*^ΔZα2/ΔZα2^ BMDMs (Fig. 3*A*, Fig. S3A). These findings suggest that the Zα2 domain of ZBP1 is essential for inflammasome activation and the induction of pyroptosis in response to fungal pathogens. In addition, both the *Zbp1*^–/–^ and *Zbp1*^ΔZα2/ΔZα2^ BMDMs showed similarly reduced activation of the markers of apoptosis (CASP8, CASP3, and CASP7) and necroptosis (pMLKL) (Fig. 3*B*, *C*, Fig. S3A). Overall, our results suggest that the Zα2 domain of ZBP1 is critical to trigger PANoptosis during fungal infection.

Given the clear role for ZBP1 in *C. albicans*- or *A. fumigatus*-mediated inflammasome activation and PANoptosis, we assessed the release of the inflammatory cytokine IL-18 after fungal infection. WT BMDMs had increased secretion of IL-18 following infection with *C. albicans* or *A. fumigatus* (Fig. 3*D*, Fig. S3B), whereas the *Zbp1*^–/–^ and *Zbp1*^ΔZα2/ΔZα2^ BMDMs had significantly reduced IL-18 release compared with the WT (Fig. 3*D*, Fig. S3B). The IL-18 release data further support that there is dampened inflammasome activation in BMDMs lacking *Zbp1*^–/–^ and *Zbp1*^ΔZα2/ΔZα2^ compared with WT BMDMs. These observations suggest that ZBP1, specifically the Zα2 domain, plays a crucial role in inducing inflammasome activation and PANoptosis in response to fungal pathogens. These results also suggest that ZBP1 senses fungal pathogens, including *C. albicans* and *A. fumigatus*, and may form a PANoptosome complex similar to that observed during bacterial and viral infections along with RIPK1, RIPK3, CASP8, CASP1, and FADD to induce PANoptosis (Fig. 3*E*).

## Discussion

In the current study, we demonstrate that the fungal pathogens *C. albicans* and *A. fumigatus* activate the ZBP1-PANoptosome, driving inflammasome activation and PANoptosis. The loss of the PANoptosome component ZBP1 or associated molecules led to inhibition of inflammasome activation, PANoptosis, and inflammatory cytokine release, and the ZBP1-Zα2 domain was crucial for these processes. PANoptosis plays a major role in host defense against pathogenic infections in addition to its functions in development and inflammatory pathophysiology (17, 19, 20, 21, 22, 23, 24, 25, 26, 27, 28). Thus far, ZBP1- and TAK1-regulated PANoptosome complexes have been identified to mediate PANoptosis (17, 25, 27, 28, 29). The dynamic composition of these PANoptosome complexes under different pathological conditions remains an active area of research.

Our study is the first to shed light on the role of ZBP1 in inflammasome activation and PANoptosis during fungal infection, and there are several interesting questions that remain to be addressed. One major question is what fungal ligands are sensed by ZBP1. Canonically, ZBP1 is known to trigger innate immune responses through the recognition of nucleic acids from viral pathogens. Fungal pathogens can liberate a variety of PAMPs including nucleic acids that could potentially serve as ligands for ZBP1 (9). It would be interesting to determine whether pure fungal ligands (such as b-glucan, zymosan, mannan) are also capable of triggering PANoptosis.

ZBP1 regulates PANoptosis and inflammasome activation during IAV infection, and genetic ablation of ZBP1 or RIPK3 and CASP8 is sufficient to rescue the host cells from IAV-induced cell death and to block inflammasome activation (20, 21). However, unlike in viral infection, deletion of ZBP1 in our fungal model did not result in a complete loss of CASP1 activation, GSDMD cleavage, or apoptotic protein (CASP8, CASP3, and CASP7) activation. Similarly, RIPK3- and RIPK3/CASP8-deficient BMDMs displayed modest reductions in the activation of pyroptosis. This complex interplay of PANoptosome components during specific infections and inflammatory ailments needs further clarification; there is likely a functional redundancy between molecules involved in the complex, which allows for key functions to be carried out even when a specific protein is lost. Previous findings suggest that it is possible that in the absence of RIPK3, ZBP1 engages the RHIM domain of RIPK1 to recruit FADD/CASP8 and potentiate pyroptosis and apoptosis (25, 26, 39, 40). The residual activation we observed in ZBP1/RIPK3-deficient BMDMs in this study suggests that there could be additional pathways or PANoptosome components such as RIPK1 that are functioning during fungal infection. In addition to NLRP3, other cytosolic sensors like AIM2 and NLRC4 can also activate inflammasomes in response to fungal pathogens. These inflammasomes could have a role in the observed residual CASP1 activation by engaging different PANoptosome complexes. Further research with double or triple knock-out cells lacking ZBP1, AIM2, NLRP3, and/or NLRC4 may address this disparity in inflammasome activation and PANoptotic cell death. RIPK1 and ZBP1 are reported to assemble PANoptosome complex in response to TAK1 inactivation and IAV infection, respectively (17, 25, 26, 27, 28). Future studies should address whether the RIPK1 PANoptosome complex is responsible for the residual inflammasome activation seen in the absence of ZBP1 during fungal *C. albicans* or *A. fumigatus* infection.

ZBP1 structurally is composed of two Zα domains in the N terminus followed by RHIM domains and a functionally uncharacterized C terminus region. RHIM domains mediate interactions with other RHIM domain-containing proteins and are crucial for regulation of cell death and inflammation. The Zα2 domain is critical in sensing nucleic acids, and we observed a critical role of the Zα2 domain in *C. albicans* or *A. fumigatus* infection to induce PANoptosis and inflammasome activation. It would be interesting to see what functions the Zα1 and RHIM domains and the C terminus region of ZBP1 have under these conditions.

Overall, our study highlights the critical role of the ZBP1-PANoptosome in driving PANoptosis and inflammation during fungal *C. albicans* or *A. fumigatus* infection. These findings improve our understanding of the host response to fungal pathogens and provide new directions for the development of targeted therapeutics for patients with mycosis, emphasizing the potential for therapeutic modulation of the PANoptosome in infectious and inflammatory diseases.

## Experimental Procedures

### Mice

All mice used in the current study were bred at the Animal Resource Center at St. Jude Children's Research Hospital and backcrossed to the C57BL/6 background for at least 10 generations. Animal studies were conducted under protocols approved by the St. Jude Children's Research Hospital Committee on the Use and Care of Animals. WT, *Zbp1*^–/–^ (21, 41), *Zbp1*^ΔZα2/ΔZα2^ (20), *Casp1/11*^–/–^ (42), *Gsdmd*^–/–^ (43), *Asc*^–/–^ (44), *Mlkl*^–/–^ (45), *Ripk3*^–/–^ (46), *Ripk3*^–/–^*Casp8*^–/–^ (47), and *Casp1/11*^–/–^*Ripk3*^–/–^*Casp8*^–/–^ (18) mice have been described previously.

### Bone marrow-derived macrophages (BMDMs) and human peripheral blood mononuclear cells (hPBMCs)

Primary granulocyte-macrophage colony-stimulating factor (GMCSF)-derived BMDMs were cultivated for 7 days in RPMI (Cellgro, Corning, 10-040-CV) supplemented with 10% FBS (Biowest, S1620), 1% sodium pyruvate, 1% nonessential amino acids (Thermo Fisher Scientific, 11140-050), 1% penicillin and streptomycin (Thermo Fisher Scientific, 15070-063), 0.1% β-mercaptoethanol and 20 ng/ml of granulocyte-macrophage colony-stimulating factor (11). These BMDMs were then seeded into 12-well culture plates (3513, Costar) in DMEM (11995-065, Gibco) supplemented with 10% FBS and 1% penicillin and streptomycin. Primary hPBMCs derived from healthy donors were cultured in RPMI supplemented with 10% FBS and were seeded into 12-well culture plates at 1 × 10^6^ cells/well.

### Fungal culture

Malt-agar slants (2% (w/v)) were used to grow *A. fumigatus* A1160 strain. After 1 week of culture, *Aspergillus* conidia were harvested using water containing 0.1% (v/v) tween-20 (9), counted, and diluted to the desired MOI for infection. *C. albicans* (ATCC-SC5314) was grown in sebouraud dextrose (SBD) broth overnight at 28 °C in a shaking water bath. Before infection, *Candida* cultures were washed twice in 1× DPBS and enumerated using a Neubauer chamber.

### Cell stimulation/infection

For *C. albicans* or *A. fumigatus* infection, primary BMDMs were infected in DMEM supplemented with 10% FBS and 1% penicillin and streptomycin at a MOI of 1 and 5, respectively, unless otherwise specified for indicated time periods. For *C. albicans* infection, hPBMCs were infected in RPMI supplemented with 10% FBS and 1% penicillin and streptomycin at a MOI of 1 or 2.

### Immunoblot analysis

For caspase blots, primary BMDMs were lysed along with the supernatant using 50 μl caspase lysis buffer (containing 1× protease inhibitors, 1× phosphatase inhibitors, 10% NP-40 and 25 mm DTT) and 100 μl 4 × SDS loading buffer. For signaling blots, supernatants were removed and BMDMs were washed one time with PBS at the indicated time points, followed by cell lysis with RIPA buffer. SDS-PAGE electrophoresis was carried out to separate proteins on 8%−12% polyacrylamide gels. PVDF membranes were used to transfer the resolved proteins, and the blots were blocked with 5% skim milk for 1 h at room temperature. Blots were incubated with primary antibodies at 4 °C, overnight, followed by incubation with secondary HRP antibodies for 1 h at room temperature. The GE Amersham Biosciences Imager 600 was used to image the immunoblots.

The following antibodies were used: anti-caspase-1 (AdipoGen, AG-20B-0042, 1:2000), anti-caspase-3 (Cell Signaling Technologies [CST], #9662, 1:1000), anti-cleaved caspase-3 (CST, #9661, 1:1000), anti-caspase-7 (CST, #9492, 1:1000), anti-cleaved caspase-7 (CST, #9491, 1:1000), anti-caspase-8 (CST, #4927, 1:1000), anti-cleaved caspase-8 (CST, #8592, 1:1000), anti-pMLKL (CST, #37333, 1:1000), anti-GSDMD (Abcam, ab209845, 1:1000), anti-ASC (AdipoGen, AG-25B-0006, 1:2000), anti-MLKL (Abgent, AP14272b,1:1000), anti-RIPK3 (ProSci, #2283, 1:1000), anti-ZBP1 (Adipogen, AG-20B-0010, 1:1000), anti-GAPDH (CST, 5174, 1:5000), and HRP-conjugated secondary antibodies (Jackson ImmunoResearch Laboratories, anti-rabbit [111-035-047], 1:5000; anti-rat [112-095-003], 1:5000; and anti-mouse [315-035-047], 1:5000).

### Real-time cell death analysis

Real-time cell death analysis was performed as previously described (17, 26). In brief, hPBMCs were seeded in 12-well plates (1 × 10^6^ cells/well) and infected with *C. albicans*. Nuclei were stained using 20 nm SYTOX Green (Thermo Fisher Scientific, S7020). Images were analyzed using IncuCyte S3 software.

### IL-18 ELISA

Inflammatory cytokine IL-18 levels were assessed using the IL-18 ELISA kit from Invitrogen (BMS618-3) following the manufacturer's protocol.

### Statistical analysis

Data analysis was performed using GraphPad Prism v8.0 software. Data are represented as mean ± S.E. Statistical significance was determined by unpaired *t* test (two-tailed) with Welch's correction for two groups and 2-way ANOVA with Sidak's multiple comparisons test for three groups. The *P* values *p* < 0.05 were statistically significant.

## Data availability

All data generated for this study are included within this manuscript.
