# Supplementary material for: ZBP1 promotes fungi-induced inflammasome activation and pyroptosis, apoptosis, and necroptosis (PANoptosis)
Source: J Biol Chem. 2021 Jan 13;295(52):18276–83. doi: 10.1074/jbc.RA120.015924 (PMC7939383; doi:10.1074/jbc.RA120.015924)
Supplement: Supplementary file 1 [file mmc1.pdf]

Supplementary Figures

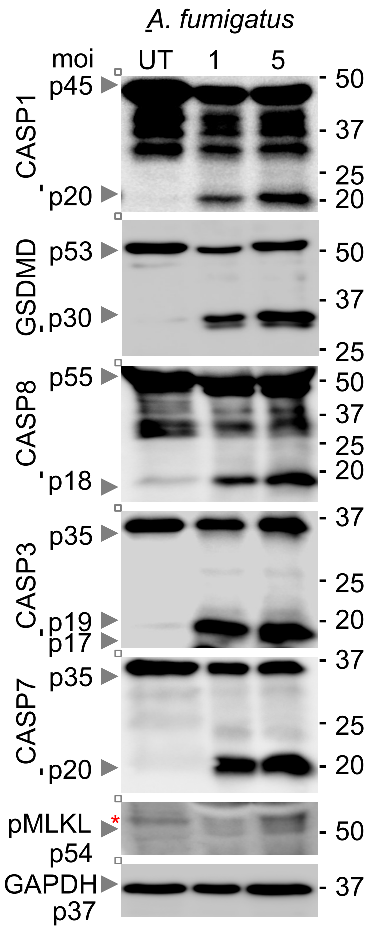

Supplementary Figure 1. PANoptosis in response to *A. fumigatus*.
Western blot analysis of PANoptosis activation markers after *A. fumigatus* infection. Pyroptosis activation is assessed by immunoblotting of cleaved caspase-1 (CASP1) (p20) and gasdermin D (GSDMD) (p30). Apoptosis activation is determined by immunoblotting of active initiator CASP8 (p18) and executioner caspases CASP3 (p19/17) and CASP7 (p20). Necroptosis activation is indicated by the phosphorylation of mixed lineage kinase domain-like pseudokinase (pMLKL). GAPDH is used as a loading control. Molecular weight marker sizes are indicated on the right (kDa). Data presented are representative of three independent experiments. moi, multiplicity of infection; UT, untreated. Red asterisks denote a non-specific band.

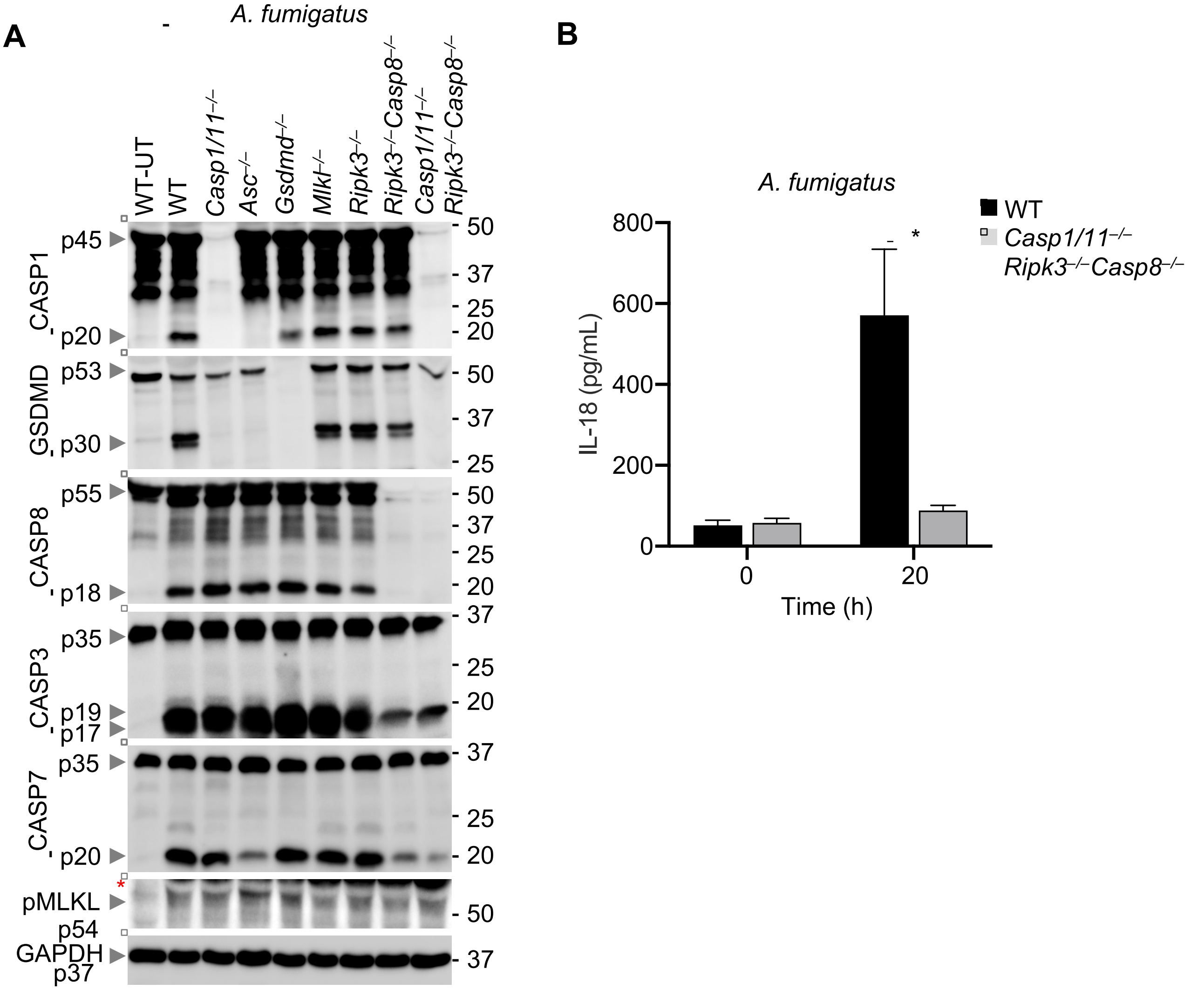

Supplementary Figure 2. Ablation of PANoptotic components inhibits *A. fumigatus*-induced PANoptosis and inflammation.

(**A**) Western blot analysis of PANoptosis activation markers after *A. fumigatus* infection in the indicated bone marrow-derived macrophages (BMDMs). Pyroptosis activation is assessed by immunoblotting of cleaved caspase-1 (CASP1) (p20) and gasdermin D (GSDMD) (p30). Apoptosis activation is determined by immunoblotting of active initiator CASP8 (p18) and executioner caspases CASP3 (p19/17) and CASP7 (p20). Necroptosis activation is indicated by the phosphorylation of mixed lineage kinase domain-like pseudokinase (pMLKL). GAPDH is used as a loading control. Molecular weight marker sizes are indicated on the right (kDa). (**B**) Inflammatory cytokine IL-18 release was evaluated in wildtype (WT) and *Casp1/11*$^{-/-}$*Rip3*$^{-/-}$*Casp8*$^{-/-}$ BMDMs following *A. fumigatus* infection for 20 h. Data shown are representative of at least three independent experiments (**A-B**). Unpaired t test with Welch's correction was used to determine statistical significance. *P < 0.05 (**D**). UT, untreated. Red asterisks denote a non-specific band.

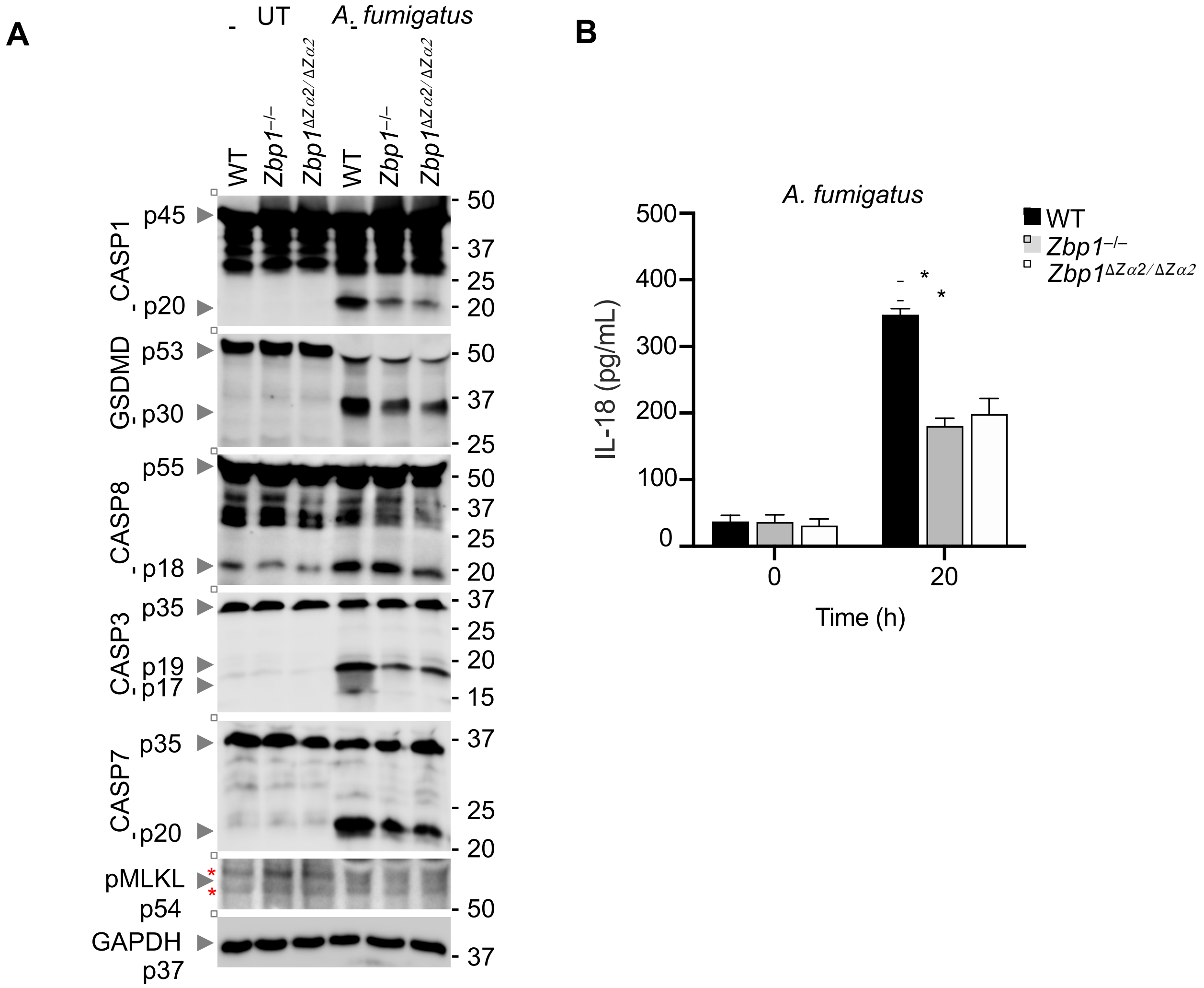

Supplementary Figure 3. Zα2 domain of ZBP1 drives *A. fumigatus*-induced PANoptosis.
Western blot analysis of PANoptosis activation markers after *A. fumigatus* infection in wildtype (WT), *Zbp1*$^{-/-}$, or *Zbp1*$^{\Delta Z\alpha2/\Delta Z\alpha2}$ bone marrow-derived macrophages (BMDMs). (**A**) Pyroptosis activation is assessed by immunoblotting of cleaved caspase-1 (CASP1) (p20) and gasdermin D (GSDMD) (p30). Apoptosis activation is determined by immunoblotting of active initiator CASP8 (p18) and executioner caspases CASP3 (p19/17) and CASP7 (p20). Necroptosis activation is indicated by the phosphorylation of mixed lineage kinase domain-like pseudokinase (pMLKL). GAPDH is used as a loading control. Molecular weight marker sizes are indicated on the right (kDa). (**B**) Inflammatory cytokine IL-18 release was evaluated in WT, *Zbp1*$^{-/-}$, or *Zbp1*$^{\Delta Z\alpha2/\Delta Z\alpha2}$ BMDMs following *A. fumigatus* infection for 20 h. Data shown are representative of at least three independent experiments (**A-B**). 2-way ANOVA was employed to determine statistical significance. *P < 0.05 (**D**). UT, untreated. Red asterisks denote a non-specific band.
